# Deficit Subsurface Drip Irrigation Improves Water Use Efficiency and Stabilizes Yield by Enhancing Subsoil Water Extraction in Winter Wheat

**DOI:** 10.3389/fpls.2020.00508

**Published:** 2020-05-06

**Authors:** Ming-Da Yang, Shah Jahan Leghari, Xiao-Kang Guan, Shou-Chen Ma, Chao-Ming Ding, Fu-Jian Mei, Li Wei, Tong-Chao Wang

**Affiliations:** ^1^Collaborative Innovation Center of Henan Grain Crops, College of Agronomy, Henan Agricultural University, Zhengzhou, China; ^2^College of Land Science and Technology, China Agricultural University, Beijing, China; ^3^Field Scientific Observation and Research Base of Land Use, Ministry of Land and Resources, Henan Polytechnic University, Jiaozuo, China

**Keywords:** deficit irrigation, winter wheat, water use efficiency, physiological characteristics, population characteristics, soil water extraction

## Abstract

Understanding the temporal and spatial patterns of soil water extraction and their impacts on growth response of winter wheat to deficit subsurface drip irrigation (SDI) conditions is critical for managing water scarcity and stabilizing yield. A field experiment was conducted from 2016 to 2018 involving five SDI amounts: 0.25, 0.4, 0.6, 0.8, and 1.0 ETc, representing 25, 40, 60, 80, and 100% of crop evapotranspiration (ETc), respectively. The results showed that the 0.6 ETc treatment significantly increased soil water extraction from 40–80 and 80–140-cm from jointing to maturity as compared to the 1.0 ETc treatment. Whereas the 0.8 ETc treatment significantly increased soil water extraction from 80–140-cm deep soil from flowering to maturity in the first growing season. The crop was most water-stressed under the 0.25 and 0.4 ETc treatments, thus extracted more soil water from 0–140-cm soil profile. However, both treatments exhibited minimum plant tillers, lowest leaf water content, leaf area index (LAI), photosynthetic rate (*P*_*n*_), and transpiration rate (*T*_*r*_) as well as grain yield. All these parameters, except for leaf water content, *P*_*n*_ after the flowering stage, and grain productivity, were also reduced in the 0.6 ETc treatment than the 1.0 ETc treatment. The differences between the 0.8 and 1.0 ETc treatments were minor in terms of plant height, LAI, spike number, *P*_*n*_ and *T*_*r*_, but infertile tillers were fewer in the 0.8 ETc treatment. We obtained high yield from the 0.8 ETc treatment, and the 0.6ETc treatment resulted in the highest harvest index with improved WUE than other treatments. Integrating deficit irrigation into SDI can save water in winter wheat production in water-limited regions, which can not only enhance soil water extraction from deep soil layers, but also sustained yield by stimulating crop growth. Therefore, a deficit SDI system would be used to conserve water in water-limited regions.

## Introduction

Drought negatively affects crop growth and yield. Irrigation scheduling is an effective water management for overcoming soil water deficiency and improving yield (Vories et al., 2009; Yang et al., 2019). It has been estimated that nearly 40% of the global food supply is produced by irrigation agriculture, which makes irrigation water becoming the largest single consumer of water on the earth. The shortage of irrigation water due to the competition of industry and urban consumption threatens food security worldwide (Ayars et al., 2015; Al-Ghobari and Dewidar, 2018).

It is crucially important to efficiently manage irrigation and water consumption while maintaining or preferably yield through development of technologies (Leghari et al., 2018). Different techniques have been introduced by researchers to reduce irrigation water requirements, such as by promoting soil extraction and enabling the crop to uptake majority of available stored water (Li et al., 2005). Making full use of soil water storage and increasing the proportion of soil water extraction in water consumption has been found to increase yield (Wang et al., 2006) by improving the leaf water status of plants and maintaining the transpiration rate (*T*_*r*_) (Zegada-Lizarazu and Iijima, 2005). Deep rooting is essential to water extraction from the bottom layers of the soil, and micro-irrigation and water management can be used to manipulate rooting depth (Li et al., 2005, 2018; Hao et al., 2015; Xu et al., 2016). Studies showed that drought-tolerant maize hybrids extracted more soil moisture from subsoil than shallow rooted varieties (Hao et al., 2015). Micro-irrigation (surface drip irrigation and micro-sprinkling irrigation) promotes soil water extraction in deep soil by improving root length density below the 80 cm soil layers and increases yield by up to 9.8–14.2% and improves water use efficiency (WUE) by 12.3–17.7% as compared to traditional flood irrigation (Li et al., 2018). Shallow rooting in deep soil limits the full exploitation of available soil water (Lv et al., 2009; Zhang et al., 2009). Therefore, promoting root penetration through effective and reasonable means (e.g., new irrigation methods or appropriate water management) can increase soil water extraction from deep soil and reduce irrigation water requirements. Water condition also directly affects the growth direction of root tips because of the hydrotropism of roots (Gao et al., 2018). Subsurface drip irrigation (SDI), involving burying the drip tape in a field below the tillage layer, caused much higher soil water content in the subsoil than in the top soil (Ayars et al., 2015). A deep rooting pattern would thus be induced for extracting deep soil water and soil water utilization was improved. Romero et al. (2004) found that SDI, especially deficit SDI, could produce a larger horizontal distribution of fine roots in the soil profile and stimulate a deeper root development than surface drip irrigation. SDI has better and more stable soil water conditions in the middle and deep soil layer, improving WUE, when compared to surface drip irrigation (Yang et al., 2019). Nevertheless, it is not clear whether SDI has better water conditions in the lower soil layer and actively induces root penetration to help promote the utilization and extraction of soil water.

Soil water availability significantly impacts photosynthesis and the morphological characteristics of plants, thus affecting crop yield and water use (Wu and Bao, 2015; Jha et al., 2017, 2019; Yang et al., 2019). Drip emitters of the SDI system are placed below the soil surface to conserve water and minimize evaporative losses. This approach has great potential to reduce irrigation water requirements and increase WUE. By increasing water availability in lower depths of the soil profile, SDI was found to, increase the photosynthetic rate (*P*_*n*_), transpiration rate (*T*_*r*_), and light energy utilization efficiency, and significantly increased the yield (Tao et al., 2015). In addition, the leaf area index is higher after the silking stage in SDI than in DI, and dry matter accumulations after silking and the aboveground biomass of the maize are enhanced in SDI (Xu et al., 2015). However, a bigger leaf area size of the plant is not necessarily better. Although increasing the water supply is beneficial to increase the leaf area index (Sadras et al., 1993), a large leaf area is associated with higher *T*_*r*_ intensity, which reduces WUE (Xu et al., 2016). Furthermore, increases in yield may not always be obtained through the maximization of plant water uptake (Bell et al., 2018). Deficit irrigation would be an effective irrigation practice to reduce irrigation water and increase WUE, because plants can reduce their leaf transpiration and soil evaporation to minimize water consumption. However, it may lead to various physiological disorders, such as an altered root system and poor shoot growth (Rahil and Qanadillo, 2015). The real challenge then is to establish a deficit irrigation system that optimizes physiological processes and maintains or even increases crop production with reducing irrigation water (Chai et al., 2016). For that, deficit irrigation requires precise knowledge of the crop growth and yield response to the water applied (Fereres and Soriano, 2007). Deficit SDI has been studied in vegetables and fruits (Badr et al., 2010; Sharma et al., 2014; Al-Ghobari and Dewidar, 2018; Çolak et al., 2018), but the information related to cereal crops is limited, particularly regarding the impact of deficit SDI on winter wheat in the region of Huang-Huai-Hai Plain (3HP).

Huang-Huai-Hai Plain is one of the most important food production areas in China (Ma et al., 2019), where winter wheat is widely (Liu et al., 2011). With the increasing water shortage, the effect of flood, sprinkler, and drip irrigation methods on winter wheat is mostly investigated (Liu et al., 2011, 2013; Xu et al., 2016; Ma et al., 2019), and all still produce poor WUE. SDI technique offers water savings and yield improvement, which makes them a better choice to solve the problem of water shortages (Lamm and Trooien, 2003; Badr et al., 2010; Ayars et al., 2015). Therefore, research about the SDI system on field crops such as winter wheat and summer maize is essential and holds great promise in this region (Yu et al., 2010; Gao et al., 2014).

The objectives of this study were to: (1) evaluate the soil water extraction and soil water dynamics under SDI; and (2) to explore the effects of SDI on morpho-physiological traits and yield in winter wheat.

## Materials and Methods

### Experimental Site

**FIGURE 1:**
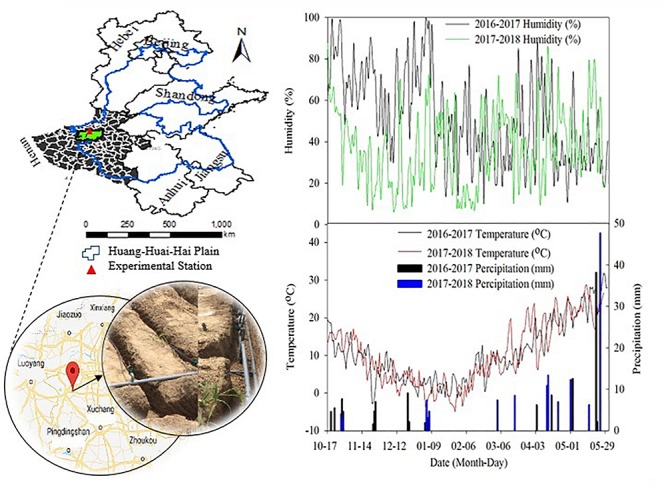
Location of experiment station and meteorological parameters of region during experimental years.

The study site was Experimental station of Henan Agricultural University, Zhengzhou, China, located at 34°47′N, 113°38′E and 70 m above sea (Figure 1).

Based on 30 years of meteorological data (1986–2015), the annual average minimum and maximum air temperature are 10.1 and 20.5°C, respectively. While, the mean precipitation is calculated as 632 mm yr^–1^ with a range of 380–991 mm and the precipitation during winter wheat growth season averages 212 mm with a range of 123–359 mm, which accounts for 18–62%. The meteorological data during experimental years is shown in Figure 1.

The soil texture of the experiment site was sandy loam. Volumetric water content at field capacity (FC) and soil bulk density (BD) varied from 0.295 to 0.32 cm^3^ cm^–3^ and 1.31 to 1.38 g cm^–3^ in the soil profile, respectively. The chemical parameters of the 0–30-cm soil layer before sowing in 2016 were as follows: available N 74.2 mg kg^–1^; Olsen-P 70.3 mg kg^–1^; exchangeable-K 229.4 mg kg^–1^; organic matter content 1.07 %. Further soil properties for deep soil layers are presented in Table 1. The soil water storage (mm) of 0–100-cm soil profile before sowing for the 0.25, 0.4, 0.6, 0.8 and 1.0 ETc treatments was 290, 282, 288, 285, and 293 mm in 2016–2017, and 262, 260, 271, 267, and 272 mm in 2017–2018, respectively.

**TABLE 1:** Main physical characteristics of the experimental site soil.

**Depth (cm)**	**Particle fraction (%)**			**Soil texture (USDA)**	**BD (g cm**^–^**^3^)**	***θs* (cm^3^ cm**^–^**^3^)**	***θ_*fc*_* (cm^3^ cm**^–^**^3^)**	***θ_*r*_* (cm^3^ cm**^–^**^3^)**	***Ks* (cm d**^–^**^1^)**
	**Sand**	**Silt**	**Clay**						
0–20	55.9	29.0	15.1	Sandy Loam	1.33	0.419	0.308	0.053	42.6
20–40	53.6	29.4	17.0	Sandy Loam	1.35	0.418	0.315	0.056	32.9
40–60	50.7	30.9	18.4	Loam	1.31	0.428	0.320	0.060	31.9
60–80	59.0	29.9	11.1	Sandy Loam	1.36	0.403	0.298	0.045	51.9
80–140	60.4	29.0	10.6	Sandy Loam	1.38	0.397	0.295	0.043	52.5

*BD, bulk density; θs, soil saturated water content; θfc, SWC at field capacity; θ_*r*_, soil residual water content; Ks, saturated hydraulic conductivity.*

Both BD and FC were determined by the cutting ring method in the laboratory (Wang et al., 2017). Soil particle fraction was analyzed by the hydrometer method (State Forestry Administration, 1999). The soil saturated, residual water content, and saturated hydraulic conductivity were obtained using Neural Network Model based on soil particle fraction and bulk density (van Genuchten, 1980).

### Experimental Design

The experience was conducted during the winter wheat growing seasons of October to May in 2016–2017 and 2017–2018. Each plot was isolated by a 14 cm width concrete wall to prevent water seepage. The size of each plot was 6.6 m^2^ (2.2 m wide × 3 m long). Five irrigation treatments were randomly assigned in a completely randomized design (CRD) includes 25, 40, 60, 80, and 100% crop evapotranspiration, denoted by 0.25, 0.4, 0.6, 0.8, and 1.0 ETc, respectively. Each treatment was replicated four times. ETc was estimated by modified FAO Penman–Monteith method (Allen et al., 1998) as follows:

(1) $$E\,T\,c=K_{c}\,\frac{0.408\,\Delta \,\left(R_{n}-G\right)+\gamma \,\left(900/T_{m\,e\,a\,n}+273\right)\,u_{2}\,\left(e_{s}-e_{a}\right)}{\Delta +\gamma \,\left(1+0.34\,u_{2}\right)}$$

Where, *ETc* is the crop evapotranspiration (mm day^–1^), *R*_*n*_ is the net radiation at the crop surface (MJ m^–2^ day^–1^), *G* is the soil heat flux density (MJ m^–2^ day^–1^), *T*_*mean*_ is the mean daily air temperature at 2 m height (°C), *u*_2_ is the wind speed at 2 m height (m s^–1^), (*e*_*s*_−*e*_*a*_) is the vapor pressure deficit (kPa), *Δ* is the slope of vapor pressure curve (kPa °C ^–1^), *γ* is the psychrometric constant (kPa °C^–1^) and *K*_*c*_ is the crop coefficient (ranged from 0.25 to 1.15 for winter wheat) which could be calibrated according to local meteorological data.

(2) $$K_{c\,m\,i\,d}=K_{c\,m\,i\,d\,\left(\text{standard}\right)}+[0.04\left(u_{2}-2\right)-0.004\left(RH_{\min}-45\right)](\frac{h}{3}){}^{0.3}$$

(3) $$K_{c\,e\,n\,d}=K_{c\,e\,n\,d\,\left(\text{standard}\right)}+[0.04\left(u_{2}-2\right)-0.004\left(RH_{\min}-45\right)](\frac{h}{3}){}^{0.3}$$

Where, *K*_*cmid*_ and *K*_*cend*_ are the calibrated crop coefficient in the middle growth stage and late growth stage, respectively, *K*_*cmid*__(stanard)_ and *K*_*cend*__(standard)_ are the standard crop coefficient (Allen et al., 1998) in the middle growth stage and late growth stage, respectively, *RH*_*min*_ is the mean daily minimum relative humidity at the measurement period (%), *h* is the crop height at the measurement period (m). When *RH*_*min*_ is not equal to 45% or *u*_2_ large or less than 2.0 m s^–1^, *K*_*c*_ can be corrected according to Eqs. (2 and 3). Data of *K*_*c*_ of winter wheat in this region during different growth periods are given in Table 2.

**TABLE 2:** Calibrated *K*_*c*_ of winter wheat during growth periods for two growing seasons.

**Growing seasons**	**Rapid growth stage**	**Middle growth stage**	**Late growth stage**
2016–2017	0.4–1.15	1.17–1.24	1.24–0.85
2017–2018	0.4–1.15	1.16–1.18	1.18–0.79

The climatic data include air temperature, relative humidity, wind speed, precipitation, and solar radiation were obtained from a meteorological station located adjacent to the experimental field and used to estimate the *ETc*.

The drip irrigation amount for each application was decided by Eq. (4).

(4) $$I=K\,\left(E\,T_{a}-P\right)$$

**FIGURE 2:**
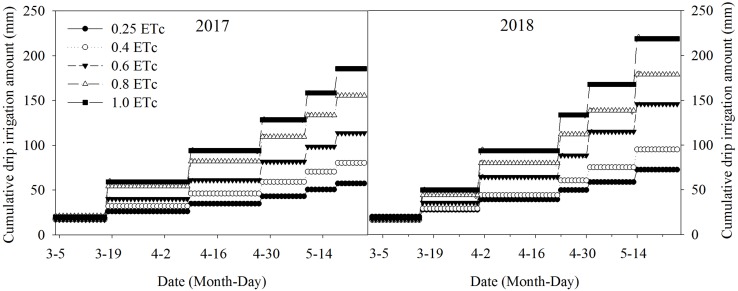
Cumulative drip irrigation amounts under different treatments during 2016–2017 and 2017–2018 growing seasons. The 0.25, 0.4, 0.6, 0.8, and 1.0 ETc represents 25, 40, 60, 80, and 100% crop evapotranspiration, respectively.

Where, *I* is the drip irrigation amount per application (mm), *ET*_*a*_ is the accumulated crop evapotranspiration during the measurement period (mm), *P* is the accumulated effective precipitation during the same duration (mm) and *K* is the coefficient used to decide the drip irrigation amount. The difference of *ET*_*a*_ and *P* provides that it should be considered for irrigation management. The *K* was set up at 0.25, 0.4, 0.6, 0.8, and 1.0 level, respectively. Based on soil water conditions, stored soil water was sufficient for winter wheat in seedling and over wintering stage. Irrigation was applied and began within the returning green stage through SDI system. When the net crop evapotranspiration value reached 35–40 mm, irrigation was started. The first irrigation was on March 4, 2017, and March 2, 2018, respectively, and all the treatments were applied the same irrigation amount (about 20 mm), because the soil water content was low after a long winter period. Afterward, the applied water was prescribed by different irrigation levels. The last irrigation was applied on May 18, 2017, and May 14, 2018, respectively. A total six drip irrigation events were performed in each growing season. The variation ranges of drip irrigation amount in the two growing seasons were 57.4–185.4 and 72.6–218.7 mm, respectively (Figure 2). The final irrigation amount percentages varied from the desired values due to inaccuracies and errors in the actual amount applied by the manual control. Actual final percentages for winter wheat were 24.2, 38.9, 59.4, 80.8, and 99.1% in 2016–2017, and 25.6, 39.0, 60.6, 79.5, and 99.0% in 2017–2018.

### Subsurface Drip Irrigation (SDI) System

Subsurface Drip Irrigation system was installed in 2015. The drip irrigation system consisted of a control unit and distribution lines. The control unit consisted of a pressure tank, a patch filter, a fertilizer applicator, and control valves. Polythene pipes of 32 mm diameter were used for the main water pipe in the system. Drip tapes were laid out connected with a capillary tube single line with separate opening valves for each subplot. The size of drip tape (Netafirm Limited Company, Tel Aviv HaShalom, Israel) was 15.9 mm inner diameter, and emitter spacing of 40 cm and had an emitter flow rate of 1.38 L H^–1^ at 100 kPa pressure. The drip tape was buried at 30 cm underground from the soil surface. The distance between two adjacent drip tapes was 60 cm, and each experimental plot had 4 laterals. The water source for irrigation was pumped from groundwater with a depth of 70 m, and the irrigation quota for each plot was controlled by a water meter.

### Crop Management

Wheat cultivar Aikang 58 was cultivated in this experiment, which was widely planted in the 3HP because of its good frost and lodging resistance. Compound fertilizer (NPK ratio 17%: 17%: 17%) was applied at the rate of 750 kg ha^–1^ before sowing. Each plot had 11 rows with a row space of 20 cm. Winter wheat was sown with a density of 225 plant m^–2^ on October 17, 2016 and October 24, 2017. Herbicides and insecticides were applied based on local farmer practice. The crop was harvested on May 28, 2017, and May 29, 2018.

### Data Collection Methods

Soil water content (SWC, cm^3^ cm^–3^) was measured at 20 cm intervals between 20 and 140 cm with a Time Domain Reflectometry (TDR) device (TRIME-PICO IPH, Germany). The measurements were performed every 15 days after sowing and every 10–15 days after the jointing stage. After irrigation and rainfall, additional measurements were taken to determine whether there was deep leakage.

Soil water storage (mm) for each soil layer was calculated from the SWC of each soil layer multiplied by the corresponding soil depth. Soil water extraction was calculated as the difference in soil water storage (0–140-cm soil profile) between the two sampling dates.

Population parameters (total tillers and ineffective tillers) were measured using a pre-selected 100 cm double-row long of each experiment plot at the late jointing and flowering stages.

All plants in the 50 cm double-row were sampled at the late jointing and flowering stages to measure the width and length of all expanding leaves. 15 plants of each experimental plot were randomly selected to measure plant height. Single leaf area = Leaf length × Leaf width × 0.83. Leaf area index (LAI) = Sum of leaf area of all expanding leaves / Land area covered by plants.

Leaf water content was measured at the late jointing stage, flowering stage, and filling stage (15 days after the flowering stage). Ten fresh leaves were weighed, and oven dried at 105°C for 0.5 h and at 70°C for 72 h or until reaching a constant weight, and then dry weight of leaves was recorded. The leaf water content (%) was calculated using the following Equation (Jin et al., 2017).

(5) Leafwatercontent(%)=(Wf-Wd)/Wf×100

Where, Wf, fresh weight and Wd, dry weight.

The flag leaf gas exchange parameters including photosynthesis (*P*_*n*_) and transpiration rates (*T*_*r*_) (Measuring the first fully expanded leaf at the top before the flowering stage) were measured using LI-6400XT with an open gas exchange system (LI-Cor, Inc., Lincoln, NE, United States) in the morning at the late jointing stage, flowering stage, and filling stage (15 days after flowering stage). 4–6 leaves were randomly selected to measure for leaf gas exchange parameters from each experiment plot.

Soil evaporation (*E*_*s*_) was represented by weight difference of consecutive measuring days after each drip irrigation event using micro-lysimeters, and the unit was converted to mm d^–1^. Each micro-lysimeters was made by PVC tubes, which consisted of inner barrel and outer barrel with the same height of 15 cm. The outer barrel with radius of 6 cm was placed between rows for each plot, which was vertically pressed into the soil with making its top surface flush with the ground, then removed soil from the barrel. The inner barrel with radius of 5.5 cm was vertically pressed into the soil and took out the inner barrel and soil, and placed the inner barrel and soil directly into the outer barrel. The bottom of the inner barrel was sealed with gauze. The inner barrel was weighted at 7:00 a.m. every morning for four consecutive days, and it was reinstalled after the next drip irrigation event. *E*_*s*_ was measured six times in each season, three times from the jointing stage to the flowering stage, and three times from the flowering stage to maturity.

After physiological maturity, grain yield and spike number were measured by harvesting approximately 1 m^2^ area in each plot and the grain weight was expressed at 13% moisture content. 30 strains of wheat in each plot were randomly selected for measuring grains spike^–1^ and 1000-grain weight (g).

Crop water use or evapotranspiration was calculated by Eq. (6).

(6) E⁢T=I+P+F-R-D+Δ⁢W

Where, *ET* is crop water use or evapotranspiration (mm), *I* is the amount of drip irrigation (mm), *P* is the amount of effective precipitation (mm), *R* is the surface runoff (mm), *D* is drainage (mm), which was calculated as *D* = (*θ*−*FC*), where *θ_*j*_* are the soil water content of the root zone (100–140-cm) in stages *j*, and *FC* is the field capacity and *D* is set to zero if *θ_*j*_*< *FC*, *F* is the capillary rise to the root zone (mm), and Δ*W* is the soil water extraction during sowing to maturity (mm). There were no capillary rise and no runoff occurred in all plots. Therefore, *ET* in this study can be calculated by Eq. (7).

(7) E⁢T=I+P-D+Δ⁢W

Water use efficiency (*WUE*, kg ha^–1^ mm^–1^) was defined by Eq. (8).

(8) W⁢U⁢E=Y/E⁢T

Where, *Y* is the grain yield (kg ha^–1^).

Radar chart analysis method was used for comprehensive evaluation. Data standardization and area of radar chart were defined by Eqs. (9 and 10), respectively (Li, 2014).

(9) $$X_{i\,j}=\frac{x_{i\,j}-x_{j\,\min}}{x_{j\,\max}-x_{j\,\min}}$$

(10) $$S_{i}=\sum_{j=1}^{p}\frac{1}{2}X_{i\,j}X_{i\,\left(j+1\right)}\sin\left(\frac{360}{p}\right){}^{\circ }$$

Where, *X*_*ij*_ is the data standardization of the *j*-th parameter under the *i*-th treatment, *x*_*ij*_ is the statistical data of the *j*-th parameter under the *i*-th treatment, *x*_*jmin*_ is the minimum value of the statistical data at the *j*-th parameter, *x*_*jmax*_ is the maximum value of the statistical data at the *j*-th parameter, *S*_*i*_ is the area of radar chart at the *i*-th treatment, *p* is number of parameters, *X*_*i*__(_*_*j*__+__1_*_)_ is the data standardization of the (*j+1*)-th parameter under the *i*-th treatment.

### Statistical Analysis

Analysis of variance (ANOVA) test was applied by using SAS (Version 8.0, SAS Inst., Cary, NC, United States) to evaluate the effect of drip irrigation treatments on the growth and yield attributes of winter wheat, WUE, ET, soil water extraction, *P*_*n*_, and *T*_*r*_. The year was not included as a factor, since drip irrigation amounts for the different treatments and precipitation varied with season. The means of each treatment were compared with the least significant difference (LSD) test at 0.05 probability level. Regression analysis was used to analyze the relationships between leaf water content, LAI, *P*_*n*_, *T*_*r*_, yield, aboveground biomass, and irrigation amount. The figures were prepared using Surfer 10 and SigmaPlot 12.5.

## Results

### Weather Parameters During Growing Seasons

Weather parameters during the two winter wheat growing seasons are shown in Figure 1. Before the turning green stage of the winter wheat (from October to February), the humidity in the 2016–2017 growing season was higher than that in 2017–2018, with an average humidity of 61.6 and 33.8%, respectively. The average humidity in March, April, and May for two growing seasons was 41.7, 44.9, 35.1% and 41.8, 41.9, and 45.0%, respectively. The temperature of winter wheat unexpectedly dropped at the early booting stage in 2017–2018, when the average temperature was 20.3°C from March 31 to April 3, but the average temperature dropped to 8.8°C from April 4 to April 7. The cumulative precipitation in the two growing seasons was 120.6 and 131.2 mm, respectively. During 2016–2017, more precipitation occurred in the earlier growing stage (57.8 mm from October to February, accounting for 47.9% of the total precipitation), whereas less precipitation occurred in the earlier growing stage in 2017–2018 (18.5 mm from October to February, accounting for 14.1% of the total precipitation). In two growing seasons, the precipitation in May was 52.7 and 66.0 mm, respectively, which was relatively abundant. But most of the precipitation occurred in the later grain-filling stage (after May 20, the precipitation was 40.2 and 47.6 mm, respectively).

### Temporal and Spatial Distribution of Soil Water Content

**FIGURE 3:**
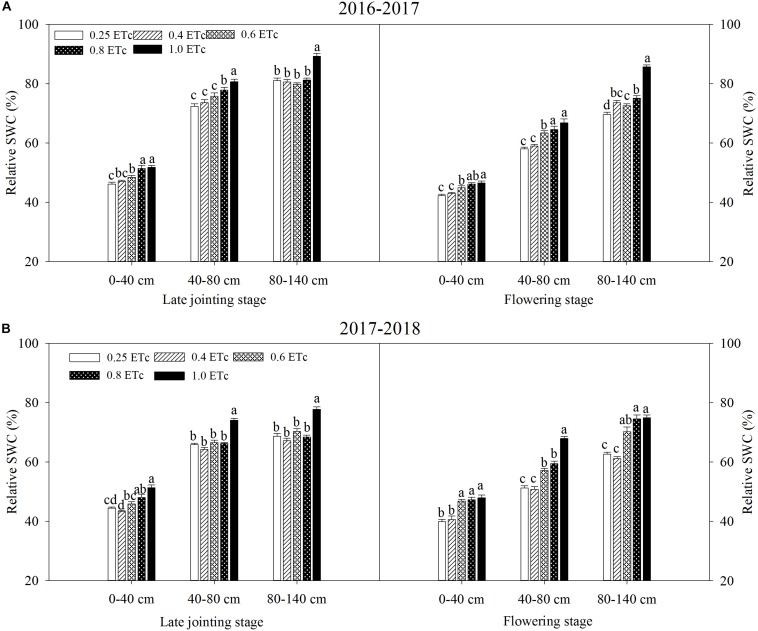
Relative soil water content at different growth stages under different drip irrigation treatments during 2016–2017 **(A)** and 2017–2018 **(B)** growing season. Vertical bars represent standard errors (*n* = 4). Different lowercase letters above the bars in the same growing stage are significantly different among different treatments at *P* < 0.05. The 0.25, 0.4, 0.6, 0.8, and 1.0 ETc represents 25, 40, 60, 80, and 100% crop evapotranspiration, respectively.

**FIGURE 4:**
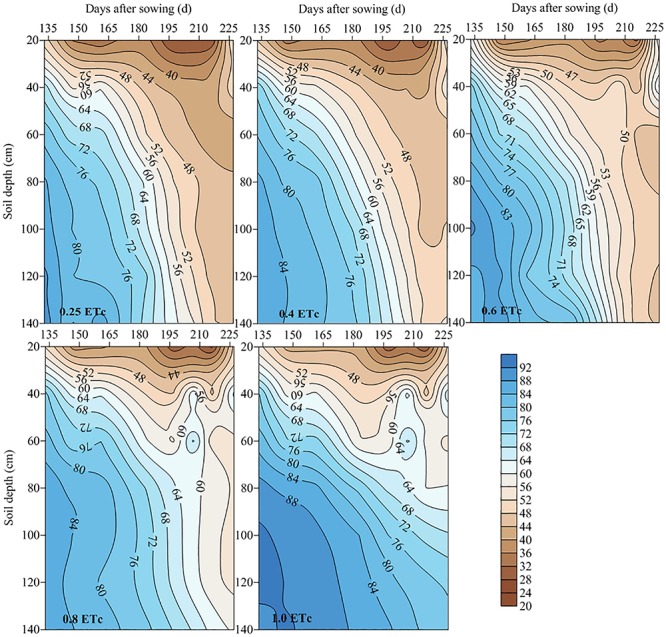
Temporal and spatial distribution of relative soil water content under different drip irrigation treatments (2016–2017). The 0.25, 0.4, 0.6, 0.8, and 1.0 ETc represents 25, 40, 60, 80, and 100% crop evapotranspiration, respectively.

**FIGURE 5:**
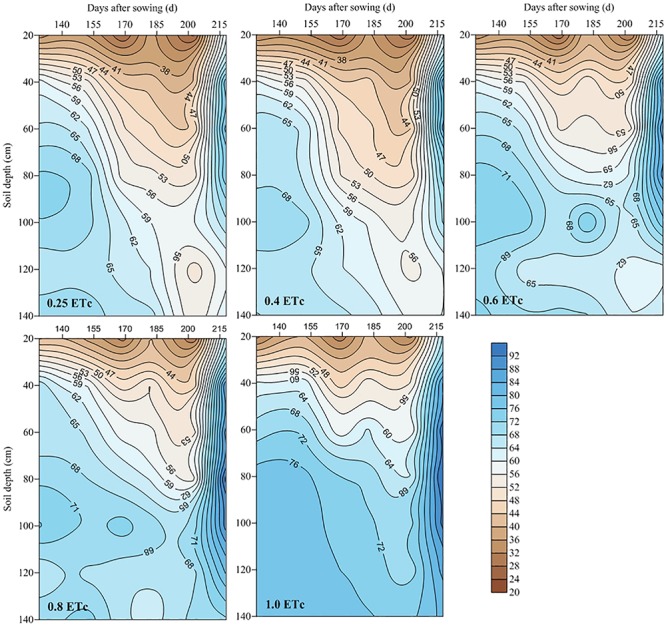
Temporal and spatial distribution of relative soil water content under different drip irrigation treatments (2017–2018). The 0.25, 0.4, 0.6, 0.8, and 1.0 ETc represents 25, 40, 60, 80, and 100% crop evapotranspiration, respectively.

We found that the 0.25 and 0.4 ETc (representing 25 and 40% of crop evapotranspiration, or ETc) treatments significantly lowered the relative SWC in the two test stages (Figure 3). At the late jointing stage, compared to the 1.0 ETc treatment, the one with 0.6 ETc significantly decreased the relative SWC of the 0–40-cm soil layer, however, the 0.8 ETc treatment had an insignificant effect on the relative SWC of the 0–40-cm soil layer when compared to the 1.0 ETc treatment. Moreover, the 0.6 and 0.8 ETc treatments significantly lowered the relative SWC of the 40–80 and 80–140-cm soil layers as compared to the 1.0 ETc treatment. At the flowering stage of the 2016–2017 growing season, there were no significant differences on the relative SWC of the 0–40 and 40–80-cm soil layers between the 0.8 and 1.0 ETc treatments. Although the 0.6 ETc treatment significantly lowered the relative SWC of the 40–80-cm soil layer, the relative SWC of the 40–80-cm soil layer of the 0.6 and 1.0 ETc treatments were more than 60% FC. Both of the 0.6 and 0.8 ETc treatments significantly lowered the relative SWC of the 40–80-cm soil layers when compared to the 1.0 ETc treatment, but the relative SWC of the 0.6 and 0.8 ETc treatments were close to 60% FC in 2017–2018. Moreover, the 0.6 and 0.8 ETc treatments did not significant affect the relative SWC of 80–140-cm soil layer in 2017–2018. The 0–40-cm soil layer was affected by both irrigation and precipitation, so the fluctuation of SWC was larger (Figures 4, 5). During the 2016–2017 growing seasons, for both the 0.25 and 0.4 ETc treatments, the average relative SWC of the 40–80-cm soil layer from the jointing to flowering stage were both less than 65% FC, and the average relative SWC of the 40–140-cm soil layers from the flowering to maturity were less than 53% FC. Under the 0.6 and 0.8 ETc treatments, the relative SWC in the 40–80-cm soil layer from the jointing to flowering stage ranged from 65 to 71% FC, and 50 to 60% FC from the flowering stage to maturity in 40–140-cm soil layers. For the 1.0 ETc treatment, the average relative SWC in the 40–80-cm soil layers exceeded 75% FC from the jointing to flowering stage, and it was close to 70% FC in the 40–140-cm soil layers from the flowering stage to maturity. During the 2017–2018 growing season, the ranges of SWC in different treatments were similar to those in the 2016–2017 growing season, except that the 0.6 and 0.8 ETc treatments had low relatively SWC of 40–80-cm soil layer (about 60% FC) during the jointing to flowering stage.

### Soil Water Extraction

During the monitoring period, the highest soil water extraction layer was the 40–80-cm from the jointing to flowering stage, and it declined to the 80–140-cm from the flowering stage to maturity (Table 3). This indicated that the middle and deeper soil layers were the main water uptake layers after the jointing stage for winter wheat with SDI. The drip irrigation amount significantly affected soil water extraction at different growth stages. From the jointing to flowering stage, the 0.6 ETc treatment significantly increased soil water extraction from all the soil layers compared to the 1.0 ETc treatment. Moreover, the 0.8 ETc treatment significantly increased soil water extraction from the 40–80 and 0–140-cm soil layers in 2017–2018 compared to the 1.0ETc treatment. Both the 0.25 and 0.4 ETc treatments did not significantly increase soil water extraction from all the soil layers compared to the 0.6 ETc treatment. From the flowering stage to maturity, in 2016–2017, there were no significant differences on soil water extraction from the 0–40 and 40–80-cm soil layers between the 1.0 and 0.6 ETc or 0.8 ETc treatments. For the 80–140-cm and the whole soil profile (0–140-cm), both the 0.6 and 0.8 ETc treatments increased soil water extraction compared to the 1.0ETc treatment. In 2017–2018, the 0.6 and 0.8 ETc treatments did not affect soil water extraction compared to the 1.0ETc treatment from all the soil layers because of heavy precipitation in the later growth stage.

**TABLE 3:** Soil water extraction (mm) from the jointing to flowering stage and the flowering to maturity for each drip irrigation treatment during 2016–2017 and 2017–2018 growing seasons.

**Treatment**	**Jointing to flowering stage**				**Flowering to maturity**			
	**0–40-cm**	**40–80-cm**	**80–140-cm**	**0–140-cm**	**0–40-cm**	**40–80-cm**	**80–140-cm**	**0–140-cm**
**2016–2017**								
0.25 ETc	4.37b	19.3a	22.1a	45.8a	−9.90a	18.5a	48.4a	57.0a
0.4 ETc	5.45ab	19.4a	17.3ab	42.1ab	−15.7a	17.3ab	41.0b	42.5b
0.6 ETc	7.12a	20.4a	21.7a	49.2a	−11.3a	13.9ab	34.3b*c*	36.8bc
0.8 ETc	5.39ab	14.0b	15.1bc	34.5bc	−13.9a	12.3ab	34.0c	33.3bc
1.0 ETc	4.19b	15.0b	10.9c	30.1c	−15.8a	12.7b	22.8d	19.6d
**2017–2018**								
0.25 ETc	6.59a	20.8a	10.0ab	37.3a	−23.5a	−12.9a	8.24a	−28.1a
0.4 ETc	6.63a	21.8a	12.7a	41.2a	−24.6a	−14.4ab	3.63a	−35.4a
0.6 ETc	6.23a	19.7ab	14.1a	40.1a	−25.0a	−22.9b*c*	−14.0b	−61.9b
0.8 ETc	−0.34b	15.4b	11.8ab	26.8b	−20.6a	−28.4c	−7.25b	−56.2b
1.0 ETc	−1.94b	9.52c	8.34b	15.9c	−23.0a	−18.9ab*c*	−9.08b	−50.9b

*Values within a column followed by different lowercase letters are significantly different among different treatments at *P* < 0.05. The 0.25, 0.4, 0.6, 0.8, and 1.0 ETc represents 25, 40, 60, 80, and 100% crop evapotranspiration, respectively.*

### Plant Population Traits

We found that plant height, LAI, and total tillers were significantly decreased, but the ineffective tillers were increased in both the 0.25 and 0.4 ETc treatments in the two growing seasons (Table 4). Moreover, the plant height, LAI, total tillers, and ineffective tillers of the 0.6 ETc treatment were significantly lower than those of the 1.0 ETc treatment in two test stages, expect for total tillers in the flowering stage in 2016–2017. In addition, the 0.8 ETc treatment decreased the LAI and plant height compared to the 1.0 ETc in the late jointing stage. However, the difference between the 0.8 and 1.0 ETc treatments was not significant. Furthermore, the 0.8 ETc treatment did not affect plant height and LAI in the flowering stage when compared to the 1.0 ETc treatment. In contrast, compared with the 1.0 ETc treatment, the 0.8 ETc treatment significantly lowered ineffective tillers in the two test stages, and the 0.8 ETc treatment significantly lowered total tillers in the late jointing stage.

**TABLE 4:** Plant height, LAI, and tiller number of winter wheat at the late jointing and flowering stages under different treatments.

**Treatment**	**Late jointing stage**				**Flowering stage**			
	**Plant height (cm)**	**LAI**	**Total tiller number (10^4^ ha^–1^)**	**Ineffective tiller number (10^4^ ha^–1^)**	**Plant height (cm)**	**LAI**	**Total tiller number (10^4^ ha^–1^)**	**Ineffective tiller number (10^4^ ha^–1^)**
**2016–2017**								
0.25 ETc	45.1 d	5.02 d	1587 b	937 a	71.2 d	5.92 d	913 c	263 a
0.4 ETc	46.3 c	6.08 c	1614 b	904 ab	73.5 c	6.94 c	947 b	237 b
0.6 ETc	47.5 bc	6.93 b	1608 b	850 b	75.0 b	7.76 b	982 ab	223 b
0.8 ETc	48.9 ab	7.26 ab	1560 b	841 b	76.9 a	8.06 ab	973 ab	215 b
1.0 ETc	49.4 a	7.82 a	1679 a	925 a	77.4 a	8.30 a	1008 a	254 a
**2017–2018**								
0.25 ETc	42.1 d	4.44 d	1314 b	841 a	70.0 d	5.03 d	666 b	194 a
0.4 ETc	42.9 d	5.35 c	1339 b	806 b	72.5 c	5.79 c	708 b	175 ab
0.6 ETc	43.5 c	6.01 b	1333 b	770 bc	74.3 b	6.56 b	694 b	133 c
0.8 ETc	45.2 ab	6.66 ab	1381 b	753 c	75.6 a	7.21 a	774 a	145 c
1.0 ETc	46.0 a	6.98 a	1464 a	857 a	75.9 a	7.35 a	799 a	190 a

*Values within a column followed by different lowercase letters are significantly different among different treatments at *P* < 0.05. The 0.25, 0.4, 0.6, 0.8, and 1.0 ETc represents 25, 40, 60, 80, and 100% crop evapotranspiration, respectively.*

### Leaf Water Content and Photosynthetic Characteristics

**FIGURE 6:**
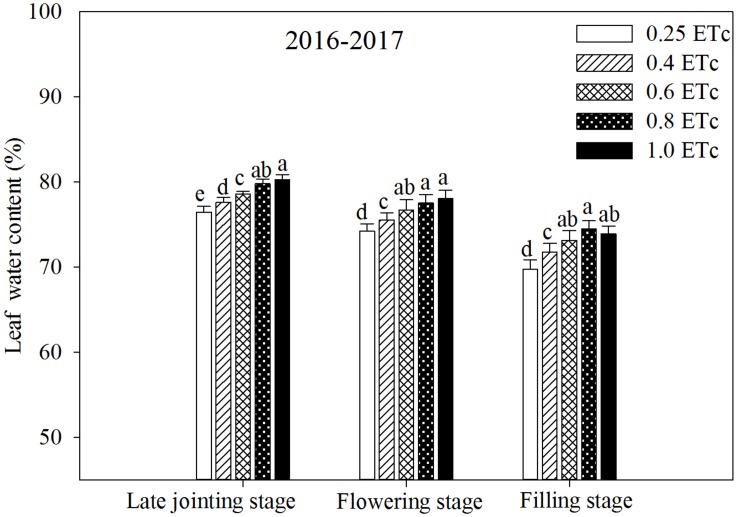
Leaf water content of winter wheat under different irrigation treatments (2016–2017). Vertical bars represent standard errors (*n* = 4). Different lowercase letters above the bars in the same growing stage are significantly different among different treatments at *P* < 0.05. The 0.25, 0.4, 0.6, 0.8, and 1.0 ETc represents 25, 40, 60, 80, and 100% crop evapotranspiration, respectively.

Compared with the 1.0ETc treatment, the 0.25 and 0.4 ETc treatments lowered leaf water content in all of the test stages (Figure 6). In addition, the 0.6 ETc treatment significantly reduced the leaf water content in the late jointing stage compared to the 1.0 ETc treatment, but the difference was not significant in the flowering and filling stages. There was no significant difference in leaf water content between the 0.8 and 1.0 ETc treatments in any test stage.

**FIGURE 7:**
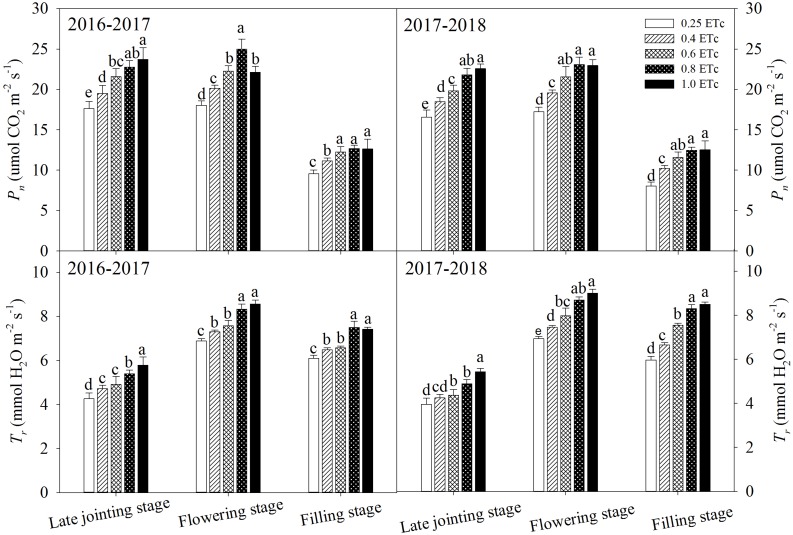
The photosynthetic rate (*P*_*n*_) and transpiration rate (*T*_*r*_) of winter wheat under different treatments. Vertical bars represent standard errors (*n* = 4). Different lowercase letters above the bars in the same growing stage are significantly different among different treatments at *P* < 0.05. The 0.25, 0.4, 0.6, 0.8, and 1.0 ETc represents 25, 40, 60, 80, and 100% crop evapotranspiration, respectively.

Furthermore, the 0.25 and 0.4 ETc treatments significantly decreased *P*_*n*_ and *T*_*r*_ in all of the test stages (Figure 7). The 0.6 ETc treatment significantly decreased *P*_*n*_ compared to the 1.0 ETc treatment in the late jointing stage but had similar *P*_*n*_ to the 1.0 ETc treatment in the flowering and filling stages. In addition, the *T*_*r*_ of the 0.6 ETc treatment was significantly lower than that of the 1.0 ETc treatment in all of the test stages. The 0.8 ETc treatment did not affect *P*_*n*_ compared to the 1.0 ETc treatment in any of the test stage in 2017–2018, and the 0.8 ETc treatment obtained the highest *P*_*n*_ in the flowering stage in 2016–2017. Compared with the 1.0 ETc treatment, the 0.8 ETc treatment significantly decreased *T*_*r*_ in late jointing stage, whereas there was no significant difference in *T*_*r*_ between the 0.8 and 1.0 ETc treatments in the flowering and filling stages.

### Yield Traits, Aboveground Biomass, and Harvest Index

The 0.25 and 0.4 ETc treatments significantly lowered the spike number, grain spike^–1^, 1000-grain weight, yield, and aboveground biomass (Table 5). Compared with the 1.0ETc treatment, the 0.6 ETc treatment significantly lowered the spike number in 2017–2018, whereas there were no significant differences in the grain spike^–1^, 1000-grain weight, and yield between the 0.6 and 1.0 ETc treatments in the two growing seasons. Moreover, the 0.6 ETc treatment decreased the aboveground biomass of winter wheat, but resulted in the highest harvest index. The harvest index of the 0.6ETc treatment was also higher than that of the 1.0 ETc treatment in 2016–2017. Compared with the 1.0 ETc treatment, the 0.8 ETc treatment significantly increased the 1000-grain weight and grain yield in 2016–2017, whereas there were no significant differences in the 1000-grain weight and grain yield between the 0.8 and 1.0 ETc treatments in 2017–2018. Furthermore, the 0.8 ETc treatment obtained the highest yield, but did not affect the aboveground biomass compared to the 1.0ETc treatment.

**TABLE 5:** Yield, yield components, aboveground biomass, and harvest index for each drip irrigation treatment during 2016–2017 and 2017–2018 growing seasons.

**Treatment**	**Spike number (10^4^ ha^–1^)**	**Grains spike^–1^**	**1000-grain weight (g)**	**Yield (kg ha^–1^)**	**Aboveground biomass (kg ha^–1^)**	**Harvest index**
**2016–2017**						
0.25 ETc	650 c	33.5 b	35.8 c	7991 d	15795 d	0.440 bc
0.4 ETc	710 b	35.6 ab	37.6 b	8727 c	17529 c	0.433 cd
0.6 ETc	758 a	36.9 a	38.9 ab	10325 ab	19412 b	0.463 a
0.8 ETc	759 a	37.2 a	39.6 a	10610 a	20306 a	0.455 ab
1.0 ETc	754 a	37.3 a	37.7 b	10088 b	20985 a	0.415 d
**2017–2018**						
0.25 ETc	473 c	27.8 c	45.6 d	6481 d	11958 e	0.471 a
0.4 ETc	533 b	31.1 b	46.9 c	7648 c	13585 d	0.480 a
0.6 ETc	562 b	34.4 a	47.2 bc	8536 b	15187 c	0.489 a
0.8 ETc	629 a	35.7 a	48.4 a	9171 a	16398 a	0.486 a
1.0 ETc	608 a	35.0 a	47.6 abc	8930 ab	16014 ab	0.485 a

*Values within a column followed by different lowercase letters are significantly different among different treatments at *P* < 0.05. The 0.25, 0.4, 0.6, 0.8, and 1.0 ETc represents 25, 40, 60, 80, and 100% crop evapotranspiration, respectively.*

### Soil Evaporation (*E*_*s*_), Soil Water Extraction From Sowing to Maturity, ET, and WUE

The 0.25 and 0.4 ETc treatments significantly increased soil water extraction from sowing to maturity, while their *E*_*s*_ and ET significantly lowered when compared to the 1.0 ETc treatment (Tables 6, 7). Furthermore, these treatments had no effect on the WUE compared to the 1.0 ETc treatment except for the 0.4 ETc treatment in 2017–2018. Compared with the 1.0 ETc treatment, the 0.6 ETc treatment significantly decreased *E*_*s*_ and ET, whereas soil water extraction from sowing to maturity and the WUE of the 0.6 ETc treatment significantly increased. Compared with the 1.0 ETc treatment, the 0.8 ETc treatment significantly reduced *E*_*s*_ and ET in 2016–2017, and the 0.8 ETc treatment did not affect *E*_*s*_ and ET in 2017–2018. Soil water extraction from sowing to maturity and the WUE of the 0.8 ETc treatment were higher than those of the 1.0 ETc treatment.

**TABLE 6:** Soil water extraction from sowing to maturity, ET, and WUE for each drip irrigation treatment during 2016–2017 and 2017–2018 growing seasons.

**Treatment**	**Soil water extraction from sowing to maturity (mm)**	**ET (mm)**	**WUE (kg ha^–1^ mm^–1^)**
**2016–2017**			
0.25 ETc	142 a	324 e	24.7b
0.4 ETc	137 a	342 d	25.5b
0.6 ETc	132 a	372 c	27.8a
0.8 ETc	116 b	397 b	26.7a
1.0 ETc	101 b	412 a	24.5bc
**2017–2018**			
0.25 ETc	74.0 a	278 c	23.3bc
0.4 ETc	58.4 ab	285 c	26.9a
0.6 ETc	32.4 c	310 b	27.6a
0.8 ETc	46.9 bc	357 a	25.7b
1.0 ETc	20.6 d	371 a	24.1c

*ET: evapotranspiration, WUE: water use efficiency. Values within a column followed by different lowercase letters are significantly different among different treatments at *P* < 0.05. The 0.25, 0.4, 0.6, 0.8, and 1.0 ETc represents 25, 40, 60, 80, and 100% crop evapotranspiration, respectively.*

**TABLE 7:** Mean daily soil evaporation (*E*_*s*_) after drip irrigation of winter wheat under different treatments (mm d**^–^**^1^).

**Treatment**	**2016–2017**		**2017–2018**	
	**From the jointing to flowering stage**	**From the flowering to maturity**	**From the jointing to flowering stage**	**From the flowering to maturity**
0.25 ETc	0.33 d	0.55 d	0.30 c	0.42 d
0.4 ETc	0.38 d	0.62 cd	0.37 c	0.54 cd
0.6 ETc	0.46 c	0.73 bc	0.44 bc	0.63 c
0.8 ETc	0.53 b	0.78 b	0.54 ab	0.75 ab
1.0 ETc	0.62 a	0.86 a	0.65 a	0.81 a

*Values within a column followed by different lowercase letters are significantly different among different treatments at *P* < 0.05. The 0.25, 0.4, 0.6, 0.8, and 1.0 ETc represents 25, 40, 60, 80, and 100% crop evapotranspiration, respectively.*

### Comprehensive Evaluation of Irrigation Management for Winter Wheat Under SDI

**FIGURE 8:**
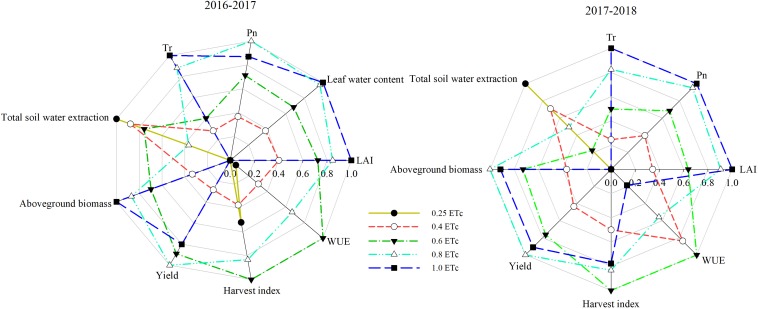
The radar chart demonstrating winter wheat performance under effect of different irrigation treatments.

A radar chart can not only qualitatively describe the merits and demerits of an evaluation object and individual parameter but also quantitatively describe the comprehensive effect of the object by calculating the area of the chart (Li, 2014). The evaluation value of the 0.8 ETc treatment on each parameter was relatively large, and the polygon formed by the scoring results expanded outward (Figure 8), indicating that guided irrigation based on 0.8 ETc was beneficial to the growth of winter wheat under SDI conditions. The 0.6 ETc treatment performed the second best. The WUE and total soil water extraction of the 1.0 ETc treatment were close to the center of the circle (Figure 8), indicating that guided irrigation based on 1.0 ETc had significant disadvantages in these aspects. The larger the area of the radar chart, the greater the advantage of the evaluation object, and the stronger its competitiveness. The area of the radar chart of the 0.8 ETc treatment was the largest, followed by the 0.6 ETc treatment, and both were larger than that of the 1.0 ETc treatment (Table 8). This indicated that guided irrigation based on 0.8 ETc or 0.6 ETc could improve coordination in the development of winter wheat under SDI conditions.

**TABLE 8:** The area of radar chart under different treatments.

**Treatment**	**2016–2017**	**2017–2018**
0.25 ETc	0.01	0.00
0.4 ETc	0.44	0.62
0.6 ETc	1.71	1.42
0.8 ETc	1.97	1.89
1.0 ETc	1.14	1.37

*Values within a column followed by different lowercase letters are significantly different among different treatments at *P* < 0.05. The 0.25, 0.4, 0.6, 0.8, and 1.0 ETc represents 25, 40, 60, 80, and 100% crop evapotranspiration, respectively.*

## Discussion

**FIGURE 9:**
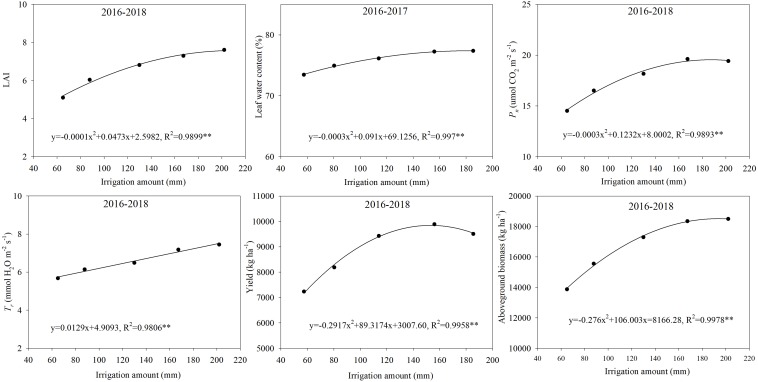
Relationships between LAI, leaf water content, *P*_*n*_, *T*_*r*_, yield, aboveground biomass, and the irrigation amount.

The enhancement in crop yield has consistently been reported to be related to the improvement of physiological characteristics, such as leaf water content, *P*_*n*_, and *T*_*r*_ (Feng and Zhou, 2018; Ma et al., 2018, 2019). Increasing irrigation not only enhances the photosynthetic physiological characteristics, but it also significantly improves the morphogenesis of plants. Jha et al. (2019) noted that winter wheat grew faster (higher plant height and LAI) under a 70% FC treatment (irrigated when SWC declined to 70% of FC) than that under 60% FC and 50% FC treatments. However, more uniform growth rate was obtained under a 60% FC treatment. Our results showed that LAI, leaf water content, *P*_*n*_, and *T*_*r*_ of plants increased significantly with an increased irrigation amount (Table 4 and Figures 6, 7). The response of LAI, leaf water content, and *P*_*n*_ to the irrigation amount fitted a quadratic model (Figure 9). These relationships were consistent with the trend between yield and the irrigation amount (Figure 9), which indicated that irrigation influences yield formation by regulating the morpho-physiological traits of plants.

Leaf water is the raw material for photosynthesis, and leaf water content can more directly reflect the actual situation of crop growth and development compared with soil water content (Feng and Zhou, 2018). The change in leaf water content has a significant impact on photosynthesis. SDI can regulate the vertical distribution of the root system in soil and induce root penetration (Romero et al., 2004), which can improve the absorption and utilization of deep soil water. Liu and Li (2005) showed that wheat could increase energy by about 0.78 J m^–2^ s^–1^ for deep roots to absorb water and improve root water absorption efficiency when the surface soil was moderately dry. Therefore, integrating deficit irrigation into SDI can synergistically improve root growth and water absorption in the subsoil, thereby regulating the physiological activity of above-ground plants. These might be the part of the reason why the 0.6 ETc treatment did not affect the physiological activity (leaf water content and *P*_*n*_) of plants compared to the 1.0 ETc treatment in the late growth period (Figures 6, 7). Nevertheless, the 0.6 ETc treatment had a significant negative effect on the morphological and physiological parameters of wheat at the late jointing stage. Winter wheat was irrigated from the jointing stage; at that time, the majority of roots were distributed in the upper soil layer. Thus, SDI with a low irrigation amount restricted the upward movement of soil water, which affected the growth of plants. A significant linear correlation was observed between the irrigation amount and *T*_*r*_ in this study (Figure 9). Combining the correlation between *P*_*n*_ and the irrigation amount (Figure 9), we could infer that *T*_*r*_ was more sensitive to irrigation than *P*_*n*_. With a decrease in the irrigation amount, the decline rate of maize *T*_*r*_ becomes higher than that of *P*_*n*_ (Li et al., 2017). Besides, since the diffusion resistance of CO_2_ is about 0.64 times that of water vapor, the reduction of stomata opening has less of an effect on *P*_*n*_ than on *T*_*r*_ (Plaut, 1995). Therefore, an appropriate reduction of stomatal conductance could significantly reduce water loss via transpiration, whereas *P*_*n*_ was not significantly affected. This might be the other reason why the 0.6 ETc treatment maintained similar physiological activity (leaf water content and *P*_*n*_) as the 1.0 ETc treatment in the late growth period. Compared with sufficient irrigation (1.0 ETc), similar physiological activities obtained by the appropriate deficit SDI (0.6 and 0.8 ETc) served as the basis for their maintenance of yields. Although the 0.6 ETc treatment significantly reduced LAI (Table 4) and could reduce photosynthetic production in leaves, non-leaf organs (e.g., ear, peduncle, and sheath) might partially compensate for the reduction in photosynthetic production in leaves at the appropriate water deficit conditions (Zhang et al., 2011).

This study showed that appropriate deficit SDI stabilized yield by optimizing population structure and reducing growth redundancy. The grain yield of a crop is correlated with the spike number, grain spike^–1^, and 1000-grain weight. The jointing stage is the critical period for deciding the sink capacity and grains per unit of the area under water-limited conditions (Royo et al., 2007; Madani et al., 2010). In this study, the 0.6 and 0.8 ETc treatments showed the non-significant effect on the spike number and grain spike^–1^, when compared to the 1.0 ETc treatment except for the 0.6 ETc treatment in 2017–2018, which could be related to the good soil water conditions from the jointing to flowering stage (averaging SWC in 40–80-cm soil layer >65% FC) (Figure 4). The shorter grain filling period in the 3HP is due to frequent dry and hot wind (Sun et al., 2006), and appropriate deficit irrigation during this period could promote grain filling. There is abundant growth redundancy in the plant height, leaf area, and tiller (or branch) number of crop, etc (Sheng, 1990), which has an undesirable impact on yield since these plant parts are key sinks for assimilates, requiring a lot of photosynthates to produce dry matter (Ma et al., 2018). The harvest index of crop population is an important indicator for measuring crop growth redundancy (Zhang et al., 1999). Therefore, reducing growth redundancy can improve yield (Sheng, 1990), and increase harvest index. In this study, the response of yield or aboveground biomass to the irrigation amount was fitted using a quadratic model (Figure 9). However, according to the quadratic model, we found that yield reached a plateau earlier than that of the aboveground biomass as the irrigation amount increased. This result indicated that a high irrigation amount could decrease the harvest index. Certainly, profitable soil water deficits can affect the distribution of photosynthetic products to different tissues and organs, thereby increasing the yield of the desired harvest and abandoning the growth of vegetative organs and the total amount of organic synthetic substances (Cai et al., 2000). According to a study by Stewart and Nielsen (1990), irrigation in the early flowering of wheat increased straw yield by 24%, but it had little effect on grain yield. The water deficit during the booting-flowering stage (SWC ranged from 60% FC to 75% FC) reduces the plant height of wheat, but delays leaf senescence and facilitates grain filling, and ultimately has an insignificant effect on yield (Wen et al., 2019). Similarly, our study showed that appropriate deficit irrigation (0.6 and 0.8 ETc) not only reduced growth redundancy (plant height, LAI, and ineffective tillers) (Table 4), but also promoted grain filling to increase the 1000-grain weight (Table 5). All the above were conducive to maintaining or even increasing yield and improving harvest index under the appropriate deficit SDI conditions (0.6 and 0.8 ETc). Moreover, we further speculated that the appropriate deficit irrigation could alleviate the impact of unfavorable weather on wheat grain filling [e.g., heavy precipitation (Figure 1)]. The 0.25 and 0.4 ETc treatments obtained the lowest yield in the present study, which were related to their low vegetative organs (e.g., LAI and plant height). Excessive reduction of growth redundancy can significantly affect crop production (Ma et al., 2018). SDI belongs to partial root-zone irrigation (Camp, 1998). Continued high soil moisture in the root zone of SDI leads to hypoxia in the root zone (Li et al., 2016), which affects crop yield and quality (Oliveira et al., 2013). Lamm and Trooien (2003) reported that irrigation with 75% crop evapotranspiration produced maximum maize yield under SDI conditions. Cotton yield plateaus when 75% or more of daily crop evapotranspiration is supplied under SDI conditions (Bhattarai et al., 2006). Our study on winter wheat under SDI showed similar results; the 1.0 ETc treatment decreased yield by 2.6–4.9% compared to the 0.8 ETc treatment. Therefore, full irrigation is not recommended for field crops under SDI conditions.

The present study indicated that appropriate deficit SDI enhanced soil water extraction in the subsoil and improved WUE. Deep water storage is vital for wheat growth because it is the primary water source in the later stage (Cui et al., 2003). Improving the soil water storage utilization of wheat, especially for deep soil water, is essential for maximizing grain yield and WUE (Ma et al., 2013; Man et al., 2015). An extra 10.5 mm of additional soil water extraction from the deep soil layer after the flowering stage increases wheat yield by 620 kg ha^–1^ (Kirkegaard et al., 2007). Song et al. (2009) found that enhancing the root ability to extract more soil water and reducing the root distribution in the topsoil layer were adaptive features for wheat under a limited water supply. Similarly, micro-irrigation increases deep soil water extraction by promoting root penetration into the deep soil layer (Li et al., 2018). As a kind of micro-irrigation, the SDI system has the greater advantage of optimizing the deep root distribution by maintaining the soil surface dryness to limit the root growth in the topsoil. The hypothesis was also verified in our study by the founding that soil water extraction came primary from middle and deep soil layers after jointing stage of wheat. Higher deep soil water extraction is related to the stronger water absorption capacity of deep roots, because the crop water availability correlates to roots system (Bengough et al., 2011). The relatively vigorous growth of winter wheat during the vegetative growth stage stimulates root growth, which subsequently enhances water utilization from soil (Zhang et al., 2013). The 0.6 and 0.8 ETc treatments with relatively good soil water conditions from the jointing to flowering stage promoted root growth. These treatments with moderate water stress during grain filling (e.g., average soil water content in the 40–140-cm soil layers ranged from 50 to 60% FC) enhanced root water uptake. Accordingly, both of the 0.6 and 0.8 ETc treatments increased soil water extraction from the deep soil layers (soil layers below 40 cm) during the grain-filling stage. Increased soil water extraction from the deep soil layers can improve morphogenesis and physiological activity of plants (such as leaf area, *T*_*r*_, stomatal conductance, and growth rate) during drought stress (Araki and Iijima, 2005; Zegada-Lizarazu and Iijima, 2005). Therefore, the appropriate deficit SDI (0.6 and 0.8 ETc) can promote the utilization of deep soil water by the root system, which is beneficial for plants to maintain their physiological activity with minimal or no impact on yield (Figures 6, 7 and Table 5). Although the 0.25 and 0.4 ETc treatments also obtained high soil water extraction from the subsoil layers (Table 3), they still showed negative effects on these parameters. This could be due to soil water extraction from the deep layer that was not sufficient to maintain the *T*_*r*_ and crop growth under severe drought conditions (Zegada-Lizarazu and Iijima, 2005). In general, the increase in deep soil water extraction is due to root penetration into deep soil, which is caused by topsoil drought (Kharrou et al., 2011). This is beneficial in reducing *E*_s_ for the deficit SDI, thus decreasing ET. All these factors combined with increasing the WUE of the 0.6 and 0.8 ETc treatments. Furthermore, a greater soil water extraction can prompt a higher soil reservoir capacity after wheat harvest and enhance downpour water penetration in the following crop season (Xu et al., 2016). Since water uptake is closely related to root distribution (Jha et al., 2017), further studies are needed to explore root traits under different drip irrigation levels within the SDI system.

The present study found that reducing non-productive water consumption (non-productive transpiration, soil evaporation, and water consumption by ineffective tillers) was important for appropriate deficit SDI to improve WUE. Crop evapotranspiration includes plant transpiration and soil evaporation (Allen et al., 2005). The primary purpose of water-saving regulation in farmland is to reduce inefficient water consumption (soil evaporation and avoid the luxury transpiration) through scientific irrigation methods and effective agronomic measures (Sun et al., 2005). There was a quadratic polynomial relationship between the irrigation amount and *P*_*n*_ or LAI, but there was a significant linear correlation with *T*_*r*_ in this study (Figure 9). This result suggested that the highest irrigation amount is not necessary because it may reduce the transpiration efficiency and leaf-level water use efficiency. The crop canopy is closely related to physiological water consumption (Slabbert and Krüger, 2014), and there is a significant positive linear correlation between LAI and crop transpiration (Zhang et al., 2014). Accordingly, compared with the 1.0 ETc treatment, the 0.6 ETc treatment decreased transpiration water loss by reducing LAI and *T*_*r*_ (Figure 7 and Table 4). Moreover, the 0.8 ETc treatment reduced non-productive transpiration water loss compared to the 1.0 ETc treatment because it reduced *T*_*r*_ at the jointing stage without significantly affecting LAI and *P*_*n*_ in any of the test stage. The full water supply can cause extra transpiration water loss (Liang et al., 2018), and significantly increase *E*_*s*_ (Table 7). Increasing productive tillers is an important method to improve wheat yield. Still, large, ineffective tillers are formed in the growth process of the wheat population, which results in growth redundancy. Sharma (1995) showed that an increase in ineffective tillers would have a negative impact on wheat production. Ineffective tillers will consume a lot of water and nutrient resources (Zhang et al., 2010). The results indicated that optimizing plant population structure and reducing ineffective tillers through appropriate agronomic measures can reduce water consumption and increase WUE and yield. Using less water to produce a similar grain yield or obtaining a higher grain yield with similar water consumption is an effective strategy to improve WUE (Zhang et al., 2017). In this study, both the 0.6 and 0.8 ETc treatments reduced ET by reducing transpiration water loss, *E*_*s*_ and water consumption of invalid tillers compared with the 1.0 ETc treatment, while obtained similar or even higher yields by optimizing population structure, thus significantly improved WUE.

Different water management models can significantly affect water, fertilizer, gas, and heat in the soil, which can influence crop morphology, physiology, yield, and water use (Li, 2014; Jha et al., 2017). Therefore, optimizing irrigation management requires careful consideration of the response of these parameters to irrigation. Previous research optimized irrigation management through regression analysis (Machado and Oliveira, 2005; Liu et al., 2011, 2013). For example, through a regression analysis of irrigation amount, yield, and WUE, Liu et al. (2011) found that 0.63 E (E represents the free surface evaporation of a 20 cm diameter pan) can be used as an irrigation strategy for winter wheat under a sprinkler irrigation in 3HP of China. Nevertheless, regression analysis cannot comprehensively evaluate multiple parameters. Some scholars have revealed that the radar chart analysis method can be used to solve the problem of comprehensive evaluation of agronomic management (Li, 2014), which was confirmed by the present study. Our study comprehensively evaluated the morpho-physiological characteristics, soil water extraction, output, harvest index, and WUE of winter wheat (Figure 8 and Table 8). The results suggested that the 0.6 and 0.8 ETc treatments can promote the coordinated growth of winter wheat, which can improve water savings, output, and efficiency.

## Conclusion

Deficit SDI increased soil water extraction from the deep soil layers. However, SDI with an extremely low irrigation water supply (0.25 ETc and 0.4 ETc) adversely affected the morpho-physiological characteristics of winter wheat because of poor soil water conditions after the jointing stage, especially during the grain-filling period. Consequently, the productivity and WUE of these treatments decreased. The 0.6 and 0.8 ETc treatments extracted an ample amount of water from the deep soil layer (80–140-cm) during the grain-filling period as compared to the 1.0 ETc treatment, and they also reduced ineffective tillers and *E*_*s*_. Moreover, the 0.6 ETc treatment reduced LAI and *T*_*r*_ except for leaf water content and *P*_*n*_ after the flowering stage when compared to the 1.0 ETc treatment. The 0.6 ETc treatment reduced ET and aboveground biomass, but it resulted similar in yield as compared to the 1.0 ETc treatment, and then the WUE and harvest index of the 0.6 ETc treatment increased. The maximum yield was obtained through the 0.8 ETc treatment because of high *P*_*n*_, *T*_*r*_, and LAI after the flowering stage. The 1.0 ETc treatment showed a yield decline because of reduced 1000-grain weight. These results indicated that SDI with proper deficit irrigation could reduce growth redundancy of plants by reducing height and effectively controlling ineffective tillers, improving water extraction from deep soil layers, and regulating physiological characteristics of plants. It can, therefore, improve WUE while maintaining yield.

## Data Availability Statement

All datasets generated for this study are included in the article/supplementary material.

## Author Contributions

M-DY and T-CW conceived and design the study. C-MD and F-JM collected the data. M-DY analyzed the data and wrote the manuscript. SL, X-KG, S-CM, and LW revised the manuscript. All authors have made good contributions to this work.

## Conflict of Interest

The authors declare that the research was conducted in the absence of any commercial or financial relationships that could be construed as a potential conflict of interest.
